# Bronchial thermoplasty reduces gas trapping in severe asthma

**DOI:** 10.1186/s12890-018-0721-6

**Published:** 2018-09-24

**Authors:** David Langton, Alvin Ing, Kim Bennetts, Wei Wang, Claude Farah, Matthew Peters, Virginia Plummer, Francis Thien

**Affiliations:** 1 Department of Thoracic Medicine, Frankston Hospital, Peninsula Health, 2 Hastings Road, Frankston, VIC 3199 Australia; 2 Faculty of Medicine, Nursing and Health Sciences, Monash University, Clayton, Vic Australia; 3 Faculty of Medicine and Health Sciences, Macquarie University, Sydney, NSW Australia; 4 Department of Thoracic Medicine, Concord Hospital, Concord, NSW Australia; 5 Sydney Medical School, University of Sydney, Sydney, NSW Australia; 6 Department of Respiratory Medicine, Eastern Health, Vic, Boxhill, Australia

**Keywords:** Bronchial thermoplasty, Severe asthma, Residual volume, Small airways dysfunction

## Abstract

**Background:**

In randomized controlled trials, bronchial thermoplasty (BT) has been proven to reduce symptoms in severe asthma, but the mechanisms by which this is achieved are uncertain as most studies have shown no improvement in spirometry. We postulated that BT might improve lung mechanics by altering airway resistance in the small airways of the lung in ways not measured by FEV_1_. This study aimed to evaluate changes in measures of gas trapping by body plethysmography.

**Methods:**

A prospective cohort of 32 consecutive patients with severe asthma who were listed for BT at two Australian university hospitals were evaluated at three time points, namely baseline, and then 6 weeks and 6 months post completion of all procedures. At each evaluation, medication usage, symptom scores (Asthma Control Questionnaire, ACQ-5) and exacerbation history were obtained, and lung function was evaluated by (i) spirometry (ii) gas diffusion (KCO) and (iii) static lung volumes by body plethysmography.

**Results:**

ACQ-5 improved from 3.0 ± 0.8 at baseline to 1.5 ± 0.9 at 6 months (mean ± SD, *p* < 0.001, paired t-test). Daily salbutamol usage improved from 8.3 ± 5.6 to 3.5 ± 4.3 puffs per day (*p* < 0.001). Oral corticosteroid requiring exacerbations reduced from 2.5 ± 2.0 in the 6 months prior to BT, to 0.6 ± 1.3 in the 6 months after BT (*p* < 0.001). The mean baseline FEV_1_ was 57.8 ± 18.9%predicted, but no changes in any spirometric parameter were observed after BT. KCO was also unaltered by BT. A significant reduction in gas trapping was observed with Residual Volume (RV) falling from 146 ± 37% predicted at baseline to 136 ± 29%predicted 6 months after BT (*p* < 0.005). Significant improvements in TLC and FRC were also observed. These changes were evident at the 6 week time period and maintained at 6 months. The change in RV was inversely correlated with the baseline FEV_1_ (*r* = 0.572, *p* = 0.001), and in patients with a baseline FEV_1_ of < 60%predicted, the RV/TLC ratio fell by 6.5 ± 8.9%.

**Conclusion:**

Bronchial thermoplasty improves gas trapping and this effect is greatest in the most severely obstructed patients. The improvement may relate to changes in the mechanical properties of small airways that are not measured with spirometry.

## Background

Bronchial thermoplasty (BT) offers an alternative therapeutic option for patients with severe asthma, defined by the Global Initiative for Asthma (GINA) as those with persistent symptoms requiring step 5 of controller treatment [1].

Performed during bronchoscopy, radiofrequency thermal impulses are delivered to airways ranging in size from 2 to 10 mm, with the intention of inducing atrophy in hypertrophied airway smooth muscle. Histological studies in both canine and humans have demonstrated that this occurs [2–5]. Three randomized controlled trials have established that patients feel better after this treatment, with fewer asthma symptoms, reduced exacerbations and improved quality of life [6–8]. However, two of these three clinical trials showed no effect of BT on the one-second forced expiratory volume (FEV_1_) [6, 7].

How is it then, that large numbers of asthmatic patients in a controlled clinical trial can experience an improvement in their symptoms and quality life, without improvement in physiological parameters such as FEV_1_? One explanation might lie in the placebo effect, known to be a powerful force in surgical treatment [9]. However, this would not explain the significantly better results observed in the active arm of a double blind, sham controlled study, namely the AIR2 trial [6]. An alternative hypothesis might be that BT leads to physiological changes which are not measured by spirometry - such as might occur in the peripheral airways.

Smaller airways, less than 2 mm in diameter, have been histologically shown to be involved in asthma [10]. These smaller airways make up a large portion of the cross-sectional area of the lung, and as a result resistance in these airways is not easily detected by changes in FEV_1_ [11, 12].

A number of methods exist to evaluate physiological changes in the small airways [13]. These include (i) plethysmography (ii) impulse oscillometry (iii) inert gas washout and (iv) sophisticated imaging techniques such as hyperpolarized magnetic resonance imaging. In this study, we report changes in plethysmographic lung volumes as measures of response to BT.

## Methods

### Participants

This was a prospective evaluation of consecutive patients selected for BT at two Australian university teaching hospitals, between June 2014 and January 2017. Participants were referred for BT by their treating respiratory physician if they had frequent symptoms despite optimized asthma therapy including high dose inhaled corticosteroids and two long acting bronchodilators. All patients were required to meet at least one of the 4 European Respiratory Society/American Thoracic Society (ERS/ATS) criteria for the definition of severe asthma, before the procedure would be considered [14].

The baseline characteristics of the patients were collated, including age, gender, body mass index (BMI), medication usage, exacerbation history, and the disease specific quality of life tool, the Asthma Control Questionnaire score (ACQ-5). The ACQ-5 was chosen as it has an established place as an evaluative tool in asthma and is known to be sensitive to change [15].

### Measurements

Lung function testing was conducted in accredited respiratory laboratories by experienced scientific staff and according to ERS/ATS standards [16], with instrument calibration immediately prior to testing. All tests were performed in the morning, and prior to the administration of any bronchodilators that day. Tests were conducted in the seated position using the Jaeger Masterscreen Body (Carefusion, Hoechberg, Germany). For spirometry, at least three acceptable maneuvers were obtained, with the FEV_1_ and the forced vital capacity (FVC) measurement values within 0.15 L of each other during repeated testing. For body plethysmography, at least three acceptable measurements were performed with functional residual capacity (FRC) values within 5% of each other. After the administration of 400mcg salbutamol, the single breath diffusing capacity (DLCO) was tested, and at least two acceptable maneuvers within 3 ml/min/mmHg of each other were required. Post bronchodilator spirometry was then performed. The predicted equations used were Quanjer [17] for spirometry, and ECCS 1993 [18] for all other tests. Testing was conducted at baseline, in the 4 weeks prior to BT being undertaken, then at 6 weeks and 6 months after the final BT procedure. Exacerbations of asthma were recorded if the patient reported a deterioration in their asthma requiring an increase in, or the commencement of, oral corticosteroids.

### Procedure

BT was performed by experienced bronchoscopists, trained in using the Alair Bronchial Thermoplasty System (Boston Scientific, NSW, Australia), using the Olympus BF-Q190 bronchoscope (Olympus Medical Systems, Tokyo, Japan) and conducted according to the previously published technique [19]. All bronchoscopies were performed under general anesthesia. Consistent with the standard protocol, each patient was treated in three sessions, three to 4 weeks apart. The right lower lobe was treated first, followed by the left lower lobe, and then both upper lobes during the final bronchoscopy. The right middle lobe was not treated. The number of radiofrequency actuations delivered was recorded for each patient. Prednisolone was prescribed for 3 days prior, and continued for 3 days after each BT procedure. All patients were electively admitted to hospital for the night immediately following treatment.

### Outcomes

The primary outcomes in this study were the changes in lung function parameters measured 6 months post procedure when compared to baseline. Secondary outcomes related to changes in ACQ-5 score, reliever and preventer medication use, and exacerbation history at 6 months. Re-evaluation at the 6 month time point was chosen so as to allow for any structural effects from BT to have been completed, yet to have avoided patients being lost to follow up or started on new medication.

### Analysis

SPSS version 24 (IBM corporation, New York, USA) was used for all statistical analyses. Grouped data refers to all 32 patients and is reported throughout as mean ± standard deviation. A paired t-test was used for paired sets of data, whilst an unpaired t-test was used to compare groups. Analysis of variance was used to compare baseline data with repeated tests over time. Pearson’s Correlation Coefficient was calculated to evaluate bivariate continuous normally distributed data. For multivariate linear regression a stepwise backward model was created. Statistical significance was taken throughout as *p* < 0.05 for a two-tailed test.

### Ethical considerations

Approval to collate and audit data as part of quality assurance was provided by the Human Research Ethics Committee at both participating institutions. All participants provided informed consent for treatment and data collection. Specific permission to use the ACQ-5 in this project was granted by its author, Elizabeth Juniper.

## Results

### Baseline characteristics

Thirty-two consecutive patients undergoing this study protocol were available for inclusion, 15 males, 17 females. No patients were lost to follow up, nor excluded. The mean age was 60.1 ± 11.7 yrs. The mean BMI was 30.4 ± 7.1 kg/m^2^. Every patient selected for treatment met the ERS/ATS definition for severe asthma, by fulfilling at least one of the four criteria. Specifically, all cases (100%) had baseline ACQ-5 scores > 1.5; 22 cases (69%) had ≥2 prednisolone courses in the previous year; and 29 cases (91%) demonstrated a baseline prebronchodilator FEV_1_ < 80% predicted. All patients had been prescribed high doses of inhaled corticosteroids, mean beclomethasone equivalent dose of 1947 ± 728 mcg daily. Sixteen patients (50%) were taking maintenance oral prednisolone, mean dose 11.0 ± 5.5 mg. All patients (100%) were taking long-acting beta_2_ agonists and long-acting muscarinic antagonists. Despite this treatment, patients used a mean of 8.3 ± 6.0 salbutamol puffs daily for rescue reliever therapy. Seven patients had been receiving stable therapy with omalizumab, for the preceding 12 months, and no patient commenced a monoclonal antibody during study period from immediately prior to BT to the 6 month re-evaluation.

The baseline prebronchodilator FEV_1_ was 57.8 ± 18.9% predicted, and the mean improvement in FEV_1_ after 400μg inhaled salbutamol was 10.9 ± 13.8%. The mean forced expiratory ratio was 53.3 ± 12.3%. The mean baseline DLCO was 85.5 ± 14.1%predicted, and the gas transfer per lung unit (KCO) was 99.5 ± 17.7%predicted. In this group of patients, twenty-four patients (75%) were never smokers, 5 patients had a pack year history of less than 10, and 3 patients had a pack year history of greater than 10. There were no current cigarette smokers.

### Procedure

The average total number of radiofrequency activations delivered per patient was 209 ± 59. No patient required treatment in the Intensive Care after the procedure, and there were no instances of prolonged hospital stay post procedure. One patient was readmitted to hospital with radiologically proven right upper lobe pneumonia 6 days after upper lobe treatment. Intravenous antibiotics were prescribed and the patient was discharged on the fourth hospital day, without further incident. One patient developed lobar collapse after BT, twice, and each time required an additional bronchoscopic procedure for suction and airway clearance.

### Outcomes

At the six-month reevaluation, the ACQ-5 had improved from 3.0 ± 0.8 to 1.5 ± 0.9 (mean difference 1.5, CI 1.1–1.9, *p* < 0.001). Only 5 patients (15.6%) did not show an improvement in ACQ-5 of greater than 0.5 units (the minimal clinically significant difference). The requirement for salbutamol rescue therapy had reduced from a mean of 8.3 ± 5.6 puffs per day to 3.5 ± 4.3 puffs per day (*p* < 0.001, paired t-test). Of 16 patients who required maintenance prednisolone pre-procedure, 12 were completely weaned from prednisolone at the 6 month follow up. A further two patients had reduced their daily prednisolone dose from 15 to 20 mg/day to 5 mg/day. The frequency of oral steroid requiring exacerbations improved from 2.5 ± 2.0 exacerbations in the 6 months prior to commencement of BT, to 0.6 ± 1.3 exacerbations in the 6 months after BT completion (*p* < 0.001, paired t- test).

### Dynamic lung function: Spirometry

Table 1 shows the effect of BT at 6 months across a range of spirometric parameters. There was no detectable effect on any variable.

**Table 1:** Dynamic Lung Function Pre and Post BT

Parameter	Baseline	6 months post	p
Prebronchodilator FEV_1_ (litres)	1.50 ± 0.54	1.50 ± 0.56	NS
Prebronchodilator FEV_1_ (%pred)	57.8 ± 18.9	58.7 ± 18.2	NS
Prebronchodilator VC (litres)	2.80 ± 0.90	2.80 ± 0.90	NS
Prebronchodilator VC (%pred)	88.2 ± 17.8	87.5 ± 18.2	NS
Prebronchodilator FEV_1_/VC (%)	53.3 ± 12.3	53.9 ± 12.4	NS
Bronchodilator response FEV_1_ (%)	10.9 ± 13.8	10.6 ± 16.0	NS
Postbronchodilator FEV_1_ (litres)	1.65 ± 0.63	1.62 ± 0.69	NS

*FEV*_*1*_ forced expiratory volume in 1 s, *VC* vital capacity, *%pred* percent predicted value

### Diffusion capacity

BT did not alter pulmonary diffusion capacity. The baseline KCO was 99.5 ± 17.7% predicted, and at the 6 month reassessment was 100 ± 15.8% predicted.

### Static lung function

Consistent with the obstructed spirometry, the static lung function tests demonstrated marked gas trapping with a mean Residual Volume (RV) of 146 ± 37% predicted. The mean RV contributed 50% of the Total Lung Capacity (TLC) (Table 2). Following BT significant improvements were observed in TLC, RV and Functional Residual Capacity (FRC). The effect size was greatest in RV where a 7% reduction was observed. The RV at 6 weeks post BT was 139 ± 38%predicted, and at 6 months post BT was 136 ± 29% predicted. Using ANOVA for repeated measures, Wilks’ Lambda was *p* = 0.002, and the multivariate partial eta squared was 0.355, indicating a strong effect. Pairwise comparisons showed the significant change occurred between baseline and 6 weeks (*p* = 0.02) after which there was no further significant change.

**Table 2:** Static Lung Function Pre bronchodilator

Parameter	Baseline	6 months	p
TLC (litres)	5.92 ± 1.42	5.73 ± 1.41	0.008
TLC (%pred)	107 ± 16	103 ± 14	0.002
RV (litres)	3.00 ± 0.99	2.80 ± 0.83	0.003
RV (%pred)	146 ± 37	136 ± 29	0.002
RV/TLC (%)	50 ± 10	49 ± 9	NS
FRC (litres)	3.72 ± 1.08	3.57 ± 1.01	0.005

*TLC* total lung capacity, *RV* residual volume, *FRC* functional residual capacity

*%pred* percent predicted value

### Subgroup analysis by airflow obstruction

**Fig. 1:**
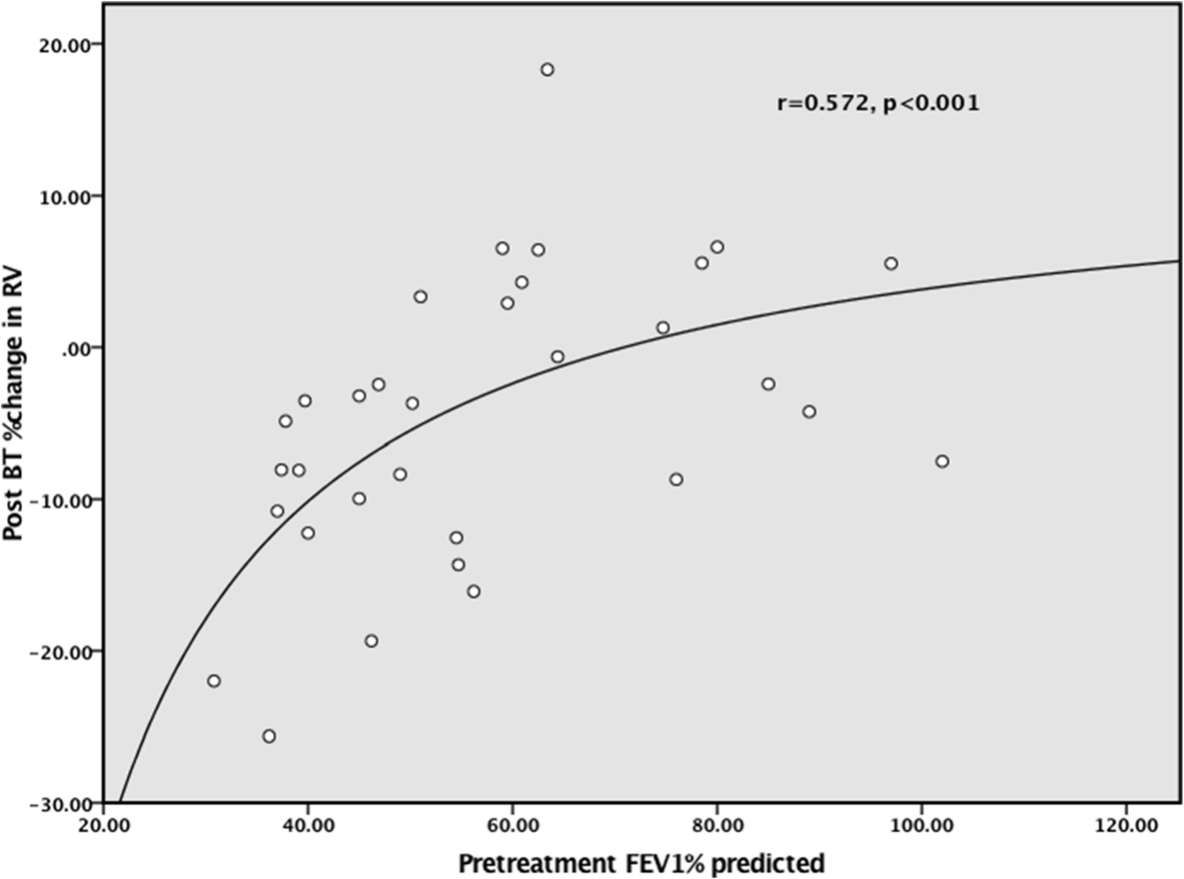
Percentage change in RV versus baseline FEV_1_% predicted

To assess whether the reduction in RV was distributed evenly across the spectrum of airflow obstruction, a scatterplot was constructed showing the percentage change in RV plotted against the baseline FEV_1_% predicted, and this is shown in Fig. 1. The graph demonstrates that the greatest improvements in RV were evident at the lower end of the baseline FEV_1_ range, with flattening of effect at the higher range of FEV_1_. The best model which described this relationship is given by the equation y = 13–930/x where y = percentage change in RV and x = FEV_1_ percent predicted, r^2^ = 0.33, *p* = 0.001.

**Fig. 2:**
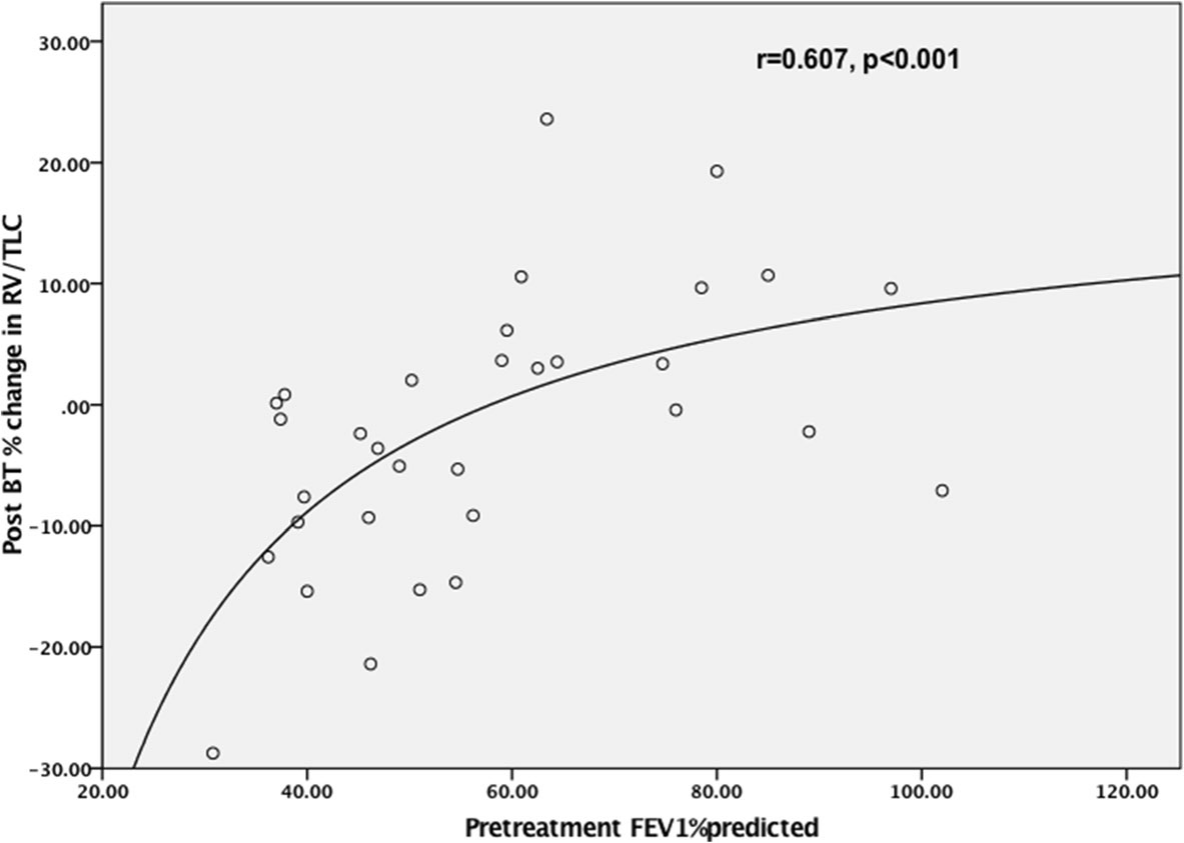
Percentage change in RV/TLC ratio versus FEV_1_% predicted

To assess whether the reduction in RV was accompanied by a reduction in RV/TLC ratio, a scatterplot was constructed showing the percentage change in the RV/TLC ratio after BT plotted against the baseline FEV_1_%predicted, and this is shown in Fig. 2. The best model to describe this relationship was given by y = 20.5–1148/x, where y = percentage change in RV/TLC ratio and x = FEV_1_ percent predicted, r^2^ = 0.37, p = 0.001.

To better understand the effect of baseline FEV_1_ on response to BT, patients were divided into two groups, around the inflection point demonstrated in Figs. 1 and 2, of baseline FEV_1_ equal to 60% predicted. These two groups are compared in Table 3. A stepwise backward multivariate linear regression model was created to examine factors predictive of percentage change in RV at 6 months post BT. The following variables had no significant effect: age, gender, baseline ACQ-5, BMI and activations. Only the baseline FEV_1_%predicted was significantly related to the change in RV (beta coefficient + 0.257, *p* = 0.002).

**Table 3:** Subgroup comparison by baseline FEV_1_

Parameter	Group A FEV_1_ < 60	Group B FEV_1_ ≥ 60	p
n	20	12	
age	61.5 ± 11.3	57.9 ± 12.5	NS
BMI kg/m^2^	30.6 ± 7.6	30.2 ± 6.5	NS
RF activations	207 ± 62	210 ± 57	NS
Baseline ACQ-5	3.0 ± 0.8	3.0 ± 0.9	NS
Baseline FEV_1_ (%predicted)	45.8 ± 8.3	77.7 ± 13.7	–
Baseline RV (litres)	3.5 ± 0.9	2.1 ± 0.4	< 0.001
Baseline RV (%predicted)	164 ± 34	114 ± 18.7	< 0.001
Baseline RV/TLC (%)	55.4 ± 8.6	42.3 ± 6.5	< 0.001
Post BT delta RV (mls)	− 326 ± 338	+ 40 ± 144	< 0.001
Post BT change RV (%)	−8.6 ± 8.4	+ 2.0 ± 7.4	< 0.001
Post BT change RV/TLC (%)	−6.5 ± 8.9	+ 7.1 ± 8.8	< 0.001

*p* unpaired t-test

## Discussion

This study recruited a group of subjects with severe asthma, persistent lung function impairment, high current symptom burden and frequent exacerbations. All were at GINA Step 5 treatment, with 50% requiring maintenance oral corticosteroids. Following BT, there was a marked improvement in current asthma control, as reflected in ACQ. Whereas no subject had an ACQ5 < 1.5 at baseline, 18/32 (56%) had achieved this at 6 months (*p* < 0.001, Chi-square). This improvement was accompanied by a 76% reduction in oral steroid requiring asthma exacerbations. Further, amongst 16 patients requiring maintenance oral corticosteroids pretreatment, 75% had been able to discontinue oral steroids by the 6-month re-evaluation.

Despite the substantive clinical improvement observed in this study, no change was seen in any spirometric parameter. This has been a consistent finding in the published literature in relation to BT [5, 6, 20, 21]. It highlights our lack of understanding of the pathophysiology of the response to BT, and underscores our desire to evaluate the effect of BT on the peripheral airways. Reassuringly, Table 1 demonstrates that BT does not attenuate the response to short acting bronchodilator- something which might otherwise have been anticipated from treatment causing atrophy of airway smooth muscle. This demonstrates that the reason that patients use less reliever medication after BT is not because the reliever medication is in any way less effective.

The absence of change in pulmonary gas diffusion following BT is also reassuring from a safety perspective. It is consistent with the normality of the lung parenchyma observed by CT scans at 5 year follow up in the AIR2 study [22], and also with reports by Thomson of diffusion capacity in the AIR trial [23].

The novel findings in this study relate to the changes in gas trapping as measured by body plethysmography. Surprisingly this aspect of lung function has not been previously reported in detail following BT, but there has been a suggestion from one CT study [24] that a reduction in total lung volume might be occurring. In the current study it is clear that BT reduces RV, and that this effect is greatest in the most obstructed patients at baseline. Accompanying the reduction in RV, a reduction in TLC and FRC are observed. The magnitude of the reduction in RV in the overall group is 7%, and this is modest but comparable to the effect of bronchodilators in this patient group. In the more severely obstructed patients, the reduction in RV is accompanied by a reduction in RV/TLC ratio. Multivariate analysis suggests that it was only the baseline FEV_1_ which was predictive of the fall in RV, with age, gender, BMI, activations and baseline ACQ-5 all having no effect. Figures 1 and 2, and Table 3 suggest that a ceiling in this effect is observed beyond a baseline FEV_1_ of 60% predicted.

Reduction in RV, without any change in spirometry, is a signal that BT may be exerting an effect in the small peripheral airways of the lung. These airways constitute a very large part of the total cross sectional area of the lung yet contribute only 10% of the total airway resistance [25]. For this reason, airways obstruction in these airways is not detected by spirometry [13]. These small airways lack the cartilaginous support of the larger airways and their premature closure leads to elevation of the RV [13]. It is well established the small airways are pathologically involved in asthma [26] and that the RV rises as the severity increases [27]. Furthermore, the increased RV is amenable to improvement with bronchodilator and anti-inflammatory therapies [28, 29]. It is entirely feasible therefore that, in this current study, the improvement in RV after BT reflects an improvement in small airways function.

Exactly how this might be occurring is open to speculation. The minimum diameter of the catheter used in BT is 1.5 mm and the bulk of BT treatment is delivered to airways greater than 2 mm in size [30]. Therefore, a mechanism must be found which would propagate the effect of BT from larger airways to small airways. It is understood that the airway smooth muscle is helically wrapped around the airways [31], and could therefore be conceptualized as acting like a coiled spring. Injury to the spring from BT would therefore weaken the apparatus along its whole length, and thus influence distal airway diameter. Alternatively, Pretolani [5] has demonstrated a marked reduction in the autonomic neural innervation of the airway following BT, and therefore it is possible that a reduction in cholinergic tone is leading to distal bronchodilatation, in the same way that targeted lung denervation is being applied in Chronic Obstructive Pulmonary Disease [32].

It is recognized that it is uncontrolled, observational data which is presented in this study. As such, its role is in hypothesis generation- in this case, about a potential new mechanism of action of BT. It is anticipated that further studies using more sensitive measures of small airways dysfunction, such as impedance oscillometry and multiple breath nitrogen washout, will be necessary to confirm the observations made and yield further insights into the role that the peripheral airways might be playing in responses to BT.

## Conclusion

The substantive clinical response to BT without any accompanying change in spirometry suggests that BT affects small peripheral airway function. Support for this concept is seen by the reduction in Residual Volume after treatment, accompanied by a reduction in RV/TLC ratio in more obstructed patients.

## Abbreviations

ACQ-5: Asthma control questionnaire-5 item version
BMI: Body Mass Index
BT: Bronchial thermoplasty
DLCO: Diffusion Capacity for carbon monoxide
ERS/ATS: European Respiratory Society/American Thoracic Society
FEV_1_: Forced expiratory volume in 1 s
FRC: Functional Residual Capacity
KCO: Gas transfer per lung unit
RV: Residual Volume
TLC: Total Lung Capacity
VC: Vital Capacity
